# Sex differences in prodromal dementia with Lewy bodies using the National Alzheimer's Coordinating Center data

**DOI:** 10.1002/alz.70275

**Published:** 2025-05-19

**Authors:** Kathryn A. Wyman‐Chick, Tanis J. Ferman, Melissa J. Armstrong, Ella A. B. Chrenka, Shannon Y. Chiu, Bhavana Patel, Matthew J. Barrett, Ece Bayram

**Affiliations:** ^1^ Department of Neurology HealthPartners Golden Valley Minnesota USA; ^2^ Department of Psychiatry and Psychology Mayo Clinic Jacksonville Florida USA; ^3^ Department of Neurology College of Medicine University of Florida Gainesville Florida USA; ^4^ Norman Fixel Institute for Neurologic Diseases University of Florida Gainesville Florida USA; ^5^ Statistical Services Health Partners Institute South Bloomington Minnesota USA; ^6^ Department of Neurology Mayo Clinic Arizona Phoenix Arizona USA; ^7^ Department of Neurology Virginia Commonwealth University Richmond Virginia USA; ^8^ Movement Disorders Center Department of Neurology University of Colorado Anschutz Aurora Colorado USA

**Keywords:** dementia, early stage, fluctuations, Lewy body disease, mild cognitive impairment, neuropsychological, Parkinsonism, precision medicine, rapid eye movement sleep behavior disorder, sex differences, visual hallucinations

## Abstract

**INTRODUCTION:**

Males are disproportionately represented in dementia with Lewy bodies (DLB). Little is known regarding sex differences in cognitive, neuropsychiatric, and motor features of the prodromal stage of DLB.

**METHODS:**

We used National Alzheimer's Coordinating Center longitudinal data to examine cognitive, neuropsychiatric, and motor features in 120 males and 23 females 2 years prior to a clinical diagnosis of DLB.

**RESULTS:**

Males were more likely than females to present with two or more core DLB clinical features 2 years prior to obtaining a diagnosis of dementia. Rapid eye movement sleep behavior disorder, cognitive fluctuations, bradykinesia, and rigidity were more common in males than females. Neuropsychological test performance was similar between groups.

**DISCUSSION:**

In prodromal DLB, males exhibit a greater number of core features than females. Core DLB features may fall short in identifying females at risk. Additional work is needed to better understand the clinical profile of prodromal DLB in females.

**Highlights:**

- There are sex differences in the presentation and course of neurodegenerative disease.
- Findings from the National Alzheimer's Coordinating Center Uniform Data Set suggest there are sex differences in prodromal DLB symptoms.
- Females were less likely to demonstrate core clinical features of DLB prior to dementia diagnosis.
- This information can help inform clinical diagnostic criteria for prodromal DLB.

## 1 INTRODUCTION

Probable dementia with Lewy bodies (DLB) is characterized by two or more core features of well‐formed visual hallucinations, cognitive fluctuations, rapid eye movement (REM) sleep behavior disorder (RBD), or Parkinsonism.^1^ However, there are well known sex differences in DLB with disproportionate representation of males,^2^ and females are less likely to experience core features at dementia onset or during disease progression.^3^, ^4^, ^5^, ^6^ Females are also more likely to exhibit Alzheimer's disease (AD) co‐pathology.^7^, ^8^ Clinical misdiagnosis with AD and delayed diagnosis of DLB are also more common for females than males.^2^ Even when females have a high likelihood for DLB phenotype based on underlying pathology without AD co‐pathology, they have a higher risk for clinical misdiagnosis with AD compared to males with similar levels of pathology.^3^, ^9^

As disease modification efforts are under way,^10^ early and accurate clinical identification is critical. Despite recent research criteria for prodromal DLB,^11^ there are limited data regarding sex‐specific symptoms that may improve the diagnostic accuracy in early‐stage disease. Large, multisite databases with prospectively collected data are ideal for investigating this research question. Thus, we aimed to investigate the sex differences in prodromal clinical presentations of DLB, leveraging longitudinal data available in the National Alzheimer's Coordinating Center Uniform Data Set (NACC‐UDS).

## 2 METHODS

### 2.1 Participants

We obtained data from the NACC‐UDS collected between September 2005 and September 2021 for participants with two or more visits up to 2 years prior to dementia diagnosis. The NACC‐UDS is a data repository aimed at collecting standardized clinical information from 39 past and present Alzheimer's Disease Research Centers (ADRCs) across the United States.^12^ The study was conducted in accordance with the ethical standards as laid down in the 1964 Declaration of Helsinki. Written informed consent was obtained from participants. The study was approved by the institutional review board at each participating ADRC data collection site.

Inclusion criteria required the absence of a dementia diagnosis for at least the first two study visits (i.e., normal cognition, cognitively impaired not mild cognitive impairment [MCI], or MCI) and a clinical diagnosis of DLB at a subsequent visit. Participants were excluded if diagnosed with Parkinson's disease and if autopsy results (if autopsy performed) did not confirm the clinical diagnosis or if there was evidence of mixed Lewy body (LB) and AD pathology.

### 2.2 Measures

We included data from the clinician report of DLB core features (visual hallucinations, cognitive fluctuations, probable RBD, and individual symptoms of parkinsonism) based on examination, as well as an interview with the participant and/or informant. For the purposes of this study, we categorized any participant as having parkinsonism if one or more criterion were present: rigidity, resting tremor, bradykinesia, parkinsonian gait disorder, postural instability.

Psychiatric symptoms were measured by the Neuropsychiatric Inventory Questionnaire (NPI‐Q).^13^ Instrumental activities of daily living were measured by the functional activities questionnaire (FAQ).^14^ A cutoff score > 6 has good clinical utility in discriminating MCI from mild dementia, with higher scores reflecting greater impairment.^15^, ^16^ Global cognition was measured using the Mini‐Mental State Examination (MMSE;^17^ NACC‐UDS Versions 1–2) or the Montreal Cognitive Assessment (MoCA;^18^ NACC‐UDS Version 3), using standard cutoff values.^19^

Neuropsychological test performances^20^ ^,^ ^21^ were normed based on age and education.^22^ NACC‐UDS Versions 1 and 2 included Wechsler Memory Scale‐Revised Digit Span (forward and backward); Trail Making Test Parts A/B; Wechsler Adult Intelligence Scale‐Revised Digit Symbol, semantic fluency; Boston Naming Test; and Wechsler Memory Scale‐Revised Logical Memory, Story A.^19^ Version 3 included Number Span (forward and backward); Trail Making Test parts A/B, letter fluency; Benson Complex Figure Copy; Multilingual Naming Test, semantic fluency; Craft Story; and Benson Complex Figure Recall.^21^

### 2.3 Statistical analysis

Demographic information, cognitive features, and clinical features were analyzed using one‐way analysis of variance or Kruskal–Wallis test for group comparisons of continuous variables, and group comparison of categorical data used the chi‐squared with Fisher exact test. Data were analyzed using SAS 9.4 analytic software. Significance was determined using a two‐sided alpha of 0.05. Pairwise evaluations included Bonferroni‐adjusted *P* values to account for multiple comparisons.[Table alz70275-tbl-0001]

## 3 RESULTS

At the first visit with a DLB diagnosis, 23 females and 120 males had one or more previous visits. Autopsy confirmation was available for 39 participants (12.8% female). Females were significantly older than males (*P *= 0.017, Table 1). There were no significant differences in global cognition, functional status, neuropsychiatric symptom severity, or the number of study visits prior to DLB diagnosis. Males were more likely than females to experience core clinical features 2 years prior to a dementia diagnosis (*P *= 0.014), but not 1 year prior or first visit with a DLB diagnosis. There were similar rates of females and males with one core clinical feature at all time points. RBD was more common in males than females 2 years prior to DLB diagnosis (*P *= 0.018) and at the first visit with a DLB diagnosis (*P *< 0.001). Cognitive fluctuations were reported in a higher percentage of males 2 years prior to dementia diagnosis (*P *= 0.027). There were no sex differences in rates of visual hallucinations at any time point (Table 2).

**RESEARCH IN CONTEXT**

1. **Systematic review**: Timely and accurate diagnosis is imperative for disease management, as well as patient and caregiver education and planning. Prodromal symptoms of dementia with Lewy bodies (DLB) have been well characterized in several cohorts; however, potential sex differences in prodromal DLB have not been thoroughly investigated.
2. **Interpretation**: Findings from the National Alzheimer's Coordinating Center Uniform Data Set suggest sex differences occur in the prodromal stage of DLB. Females were less likely to experience core clinical features, including rapid eye movement sleep behavior disorder, parkinsonism, and cognitive fluctuations. Similar rates of neuropsychiatric symptoms and neuropsychological performance were observed between groups.
3. **Future directions**: This is one of the first studies focused on sex differences in symptoms of early DLB. Several knowledge gaps remain. Future work should include biomarkers and/or neuropathology findings. This work should be replicated in global cohorts to enhance generalizability and efforts to improve approaches to precision medicine in DLB.

**TABLE 1 alz70275-tbl-0001:** Participant demographics at the time of DLB diagnosis by sex.

	Female (*N* = 23)	Male (*N* = 120)	*p* value
Age	79.0 (9.3)	74.3 (7.2)	0.017
White	89.3%	94.7%	0.28
Hispanic	7.1%	0.0%	0.002^*^
Highest education			0.20
High school	21.4%	17.4%	–
College	50.0%	35.6%	–
Graduate school	28.6%	47.0%	–
Cognitive impairment in first‐degree family member	42.9%	47.7%	0.64
Years between initial NACC visit and DLB diagnosis	2 (1, 4)	2 (1, 3)	0.11
MMSE (17 females, 65 males)	23.3 (4.3)	23.5 (5.2)	0.88
Education Adjusted MoCA (8 females, 51 males)	18.4 (5.6)	18.4 (5.3)	0.99
FAQ	17 (10, 21)	14 (9, 21)	0.62

*Note*: Values listed as mean (SD), percentage, or median (Q1, Q3).

Abbreviations: DLB, dementia with Lewy bodies; FAQ, Functional Assessment Questionnaire; MMSE, Mini‐Mental State Examination; MoCA, Montreal Cognitive Assessment; NACC, National Alzheimer's Coordinating Center.

* *P* < 0.05.

**TABLE 2 alz70275-tbl-0002:** Core clinical features preceding DLB diagnosis.

	2 years prior			1 year prior			First visit with DLB diagnosis		
Clinical features	Female (*N* = 14)	Male (*N* = 65)	*p* value	Female (*N* = 19)	Male (*N* = 95)	*p* value	Female (*N* = 23)	Male (*N* = 120)	*p* value
Parkinsonism									
Bradykinesia	35.7%	78.5%	0.003^*^	52.6%	76.8%	0.046^*^	78.3%	91.7%	0.068
Parkinsonian gait	42.9%	53.8%	0.56	57.9%	66.3%	0.599	78.3%	75.8%	>0.999
Rigidity	42.9%a	55.4%	0.556	36.8%	65.3%	0.037^*^	47.8%	69.2%	0.057
Postural instability	28.6%	9.2%	0.07	42.1%	25.3%	0.164	30.4%	26.7%	0.799
Resting tremor	21.4%	43.1%	0.227	26.3%	38.9%	0.435	30.4%	52.5%	0.069
Falls	14.3%	13.8%	>0.999	36.8%	20.0%	0.136	60.9%	30.8%	0.009^*^
Probable RBD	28.6%	64.6%	0.018^*^	36.8%	62.1%	0.073	34.8%	75.0%	<0.001^*^
Visual hallucinations	7.1%	23.1%	0.279	26.3%	26.3%	>0.999	52.2%	44.2%	0.502
Cognitive fluctuations	7.1%	40.0%	0.027^*^	21.1%	33.7%	0.418	39.1%	62.5%	0.063
Total core features									
1 core feature	78.6%	95.4%	0.065	84.2%	89.5%	0.452	100.0%	100.0%	–
2+ core features	57.1%	87.7%	0.014^*^	84.2%	82.1%	>0.999	100.0%	96.7%	>0.999

*Note*: Clinical features are based on clinician report and examination. Prior refers to 1 and 2 years prior to the DLB diagnosis visit.

Abbreviations: DLB, dementia with Lewy bodies; RBD, rapid eye movement sleep behavior disorder.

* *P* < 0.05.

A higher percentage of males than females demonstrated bradykinesia at 2 years (*P *= 0.003) and 1 year prior to DLB diagnosis (*P *= 0.046). In the year preceding a DLB diagnosis, males had higher rates of rigidity than females (*P *= 0.037). At the first visit with a DLB diagnosis, females were more likely to have falls then males (*P *= 0.009). There was no sex difference in rates of parkinsonian gait, tremors, and postural instability at any assessed period (Table 2).[Table alz70275-tbl-0002]

**FIGURE 1:**
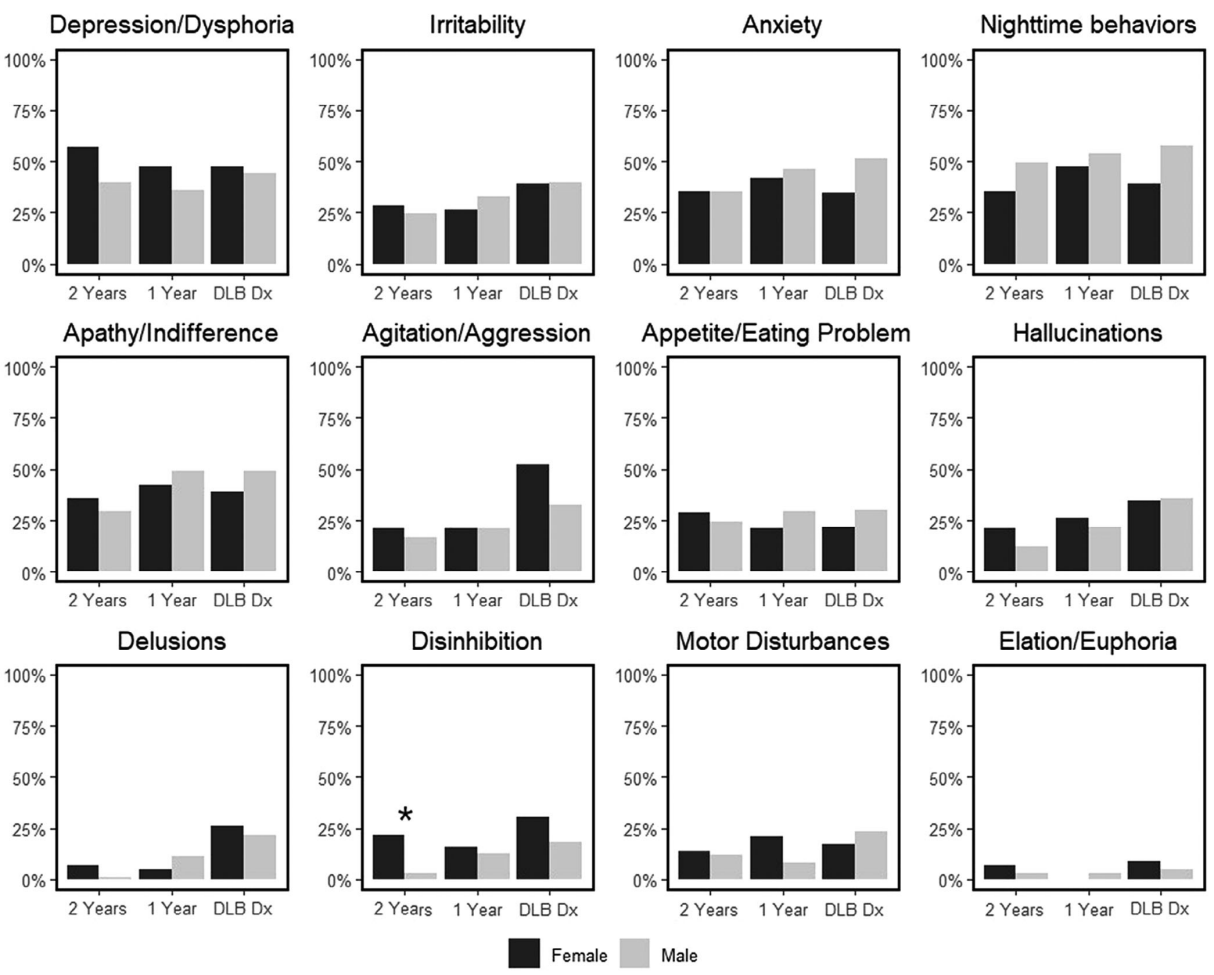
Neuropsychiatric Symptom Inventory Questionnaire (NPI‐Q) in males and females. Values represent NPI‐Q responses at visit with DLB diagnosis and at 1 and 2 years before DLB diagnosis visit. Statistically significant sex difference is marked with * for *P *< 0.05. DLB, dementia with Lewy bodies; DLB Dx, first visit with a dementia with Lewy bodies diagnosis

Individual items on the NPI‐Q did not differ between males and females (Figure 1) except for a higher percentage of females with disinhibition 2 years prior to DLB diagnosis (21% vs. 3%, *P *= 0.037), but the overall numbers were small (three females vs. two males) with questionable clinical relevance (Table S1 in supporting information). The most frequent prodromal neuropsychiatric issues included depression, apathy, anxiety, and nighttime behaviors. At the time of the first visit with a DLB diagnosis, hallucinations and aggression were commonly reported symptoms.

There were no significant sex differences in neuropsychological test performance except for significantly lower verbal fluency in males than females with the category prompt “vegetables” (Table 3; *P *= 0.004). The rate of decline on neuropsychological test measures over three time points was similar between groups (Table 3).

**TABLE 3 alz70275-tbl-0003:** Neuropsychological test performance at prodromal and DLB visits stratified by sex.

	2 years prior		1 year prior		DLB diagnosis		Group comparison	
	Female (*N* = 14)	Male (*N* = 65)	Female (*N* = 19)	Male (*N* = 95)	Female (*N* = 23)	Male (*N* = 20)	Female versus Male *p* value	Rate of change female versus male *p* value
MMSE/MoCA	−1.2 (1.4)	−1.2 (3.7)	−0.9 (1.8)	−2.2 (1.7)	−3.2 (2.6)	−2.7 (6.3)	0.631	0.690
Story recall, immediate	−1.0 (1.1)	−1.4 (1.0)	−1.0 (0.8)	−1.3 (1.1)	−1.4 (1.2)	−1.6 (1.0)	0.368	0.761
Story recall, delayed	−1.0 (1.0)	−1.3 (1.0)	−0.9 (0.9)	−1.4 (1.0)	−1.3 (1.1)	−1.6 (0.9)	0.433	0.721
Naming test	−0.4 (1.0)	−0.4 (1.2)	−0.6 (0.9)	−0.5 (1.1)	−1.2 (1.6)	−0.8 (1.5)	0.727	0.297
Trail Making Test Part A	−0.7 (1.0)	−1.8 (2.0)	−2.5 (3.1)	−2.4 (2.5)	−4.7 (2.8)	−4.0 (3.4)	0.225	0.122
Trail Making Test Part B	−2.0 (1.9)	−2.3 (2.0)	−2.9 (2.1)	−2.7 (1.9)	−3.4 (1.4)	−3.3 (1.9)	0.921	0.768
Symbol digit coding	−1.4 (0.9)	−1.8 (1.0)	−1.8 (0.8)	−2.1 (1.0)	−2.5 (1.0)	−2.5 (1.1)	0.051	0.102
Number span forward	−0.5 (1.0)	−0.3 (1.0)	0.0 (1.1)	−0.5 (1.0)	−0.5 (1.0)	−0.8 (1.1)	0.900	0.414
Longest span forward	−0.3 (0.7)	−0.1 (0.9)	0.0 (1.1)	−0.4 (1.0)	−0.3 (1.0)	−0.6 (1.1)	0.870	0.274
Number span backward	−0.8 (1.1)	−0.7 (1.0)	−0.9 (0.9)	−1.0 (0.8)	−1.5 (0.9)	−1.1 (0.9)	0.302	0.080
Longest span backward	−0.8 (0.9)	−0.8 (1.0)	−1.0 (0.8)	−1.1 (0.9)	−1.5 (1.0)	−1.1 (1.0)	0.261	0.093
Benson figure, copy	0.3 (0.8)	−0.5 (1.5)	−2.1 (3.1)	−0.8 (1.4)	−2.0 (2.7)	−2.2 (3.0)	0.867	0.945
Benson figure immediate recall	0.4 (0.1)	−0.4 (1.4)	−0.3 (1.6)	−0.4 (1.5)	−0.6 (1.7)	−0.7 (1.6)	0.556	0.707
Benson figure, delayed recall	−0.6 (2.0)	−1.4 (1.1)	−0.6 (1.9)	−1.0 (1.1)	−1.7 (1.2)	−1.7 (1.3)	0.536	0.641
Category fluency, animals	−0.7 (1.2)	−1.0 (0.9)	−1.0 (1.0)	−1.0 (1.0)	−1.7 (0.8)	−1.5 (1.1)	0.282	0.141
Category fluency, vegetables	−0.4 (1.4)	−1.1 (1.1)	−0.4 (1.5)	−1.3 (1.0)	−1.3 (1.3)	−1.8 (0.9)	0.004^*^	0.219
Phonemic fluency, F and L total	−0.4 (0.7)	−0.5 (1.1)	−0.9 (0.5)	−0.8 (1.1)	−0.9 (1.1)	−0.9 (1.3)	0.620	0.496

*Note*: Values indicate mean *z* scores (SD). *P* values obtained from mixed model (score = group + year + group x year) with random intercept to account for repeated measures within person.

Abbreviations: DLB, dementia with Lewy bodies; MMSE, Mini‐Mental State Examination; MoCA, Montreal Cognitive Assessment; Story–Immediate, Craft immediate recall or Logical Memory I; Story–Delayed, Craft delayed recall or Logical Memory II; Naming Test, Boston Naming Test or Multilingual Naming Test; Digit Span‐F, Digit Span Forward; Digit Span‐B, Digit Span Backward.

* *P* < 0.05

## 4 DISCUSSION

In this study, we examined whether sex differences were evident in the clinical presentation of the prodromal phase of DLB. In this well‐characterized North American cohort, females were less likely to experience two or more core clinical features than men 2 years prior to first visit resulting in a DLB diagnosis despite similar cognitive performance. This is consistent with research showing females experience core DLB features later in the disease process, leading to delays in meeting clinical criteria for DLB diagnosis compared to males.^5^ Given the emphasis on core clinical features for prodromal DLB in the research diagnostic criteria,^11^, ^23^ results also suggest that females with prodromal DLB may be less likely to present with two or more core features consistent with a diagnosis of probable prodromal DLB compared to males. It is unknown whether investigating symptoms beyond core clinical features by incorporating indicative features^1^ or including biological biomarkers^11^, ^24^ would improve detection of prodromal DLB. It is possible that males and females may differ in the rates of phenoconversion, with males having a more prolonged prodromal stage than females, and further investigation is needed.

In other clinically defined cohorts, females demonstrated more severe cognitive impairment than males on global screening tests, which may be related to differing severities of AD co‐pathology.^25^, ^26^ Some studies suggest females have demonstrated better cognitive functioning and are less likely to experience dementia compared to males with similar levels of LB pathology;^6^ however, our analysis in a clinically defined cohort suggested a similar pattern of cognitive performance within the prodromal phase. Males had a slower rate of verbal fluency for vegetables but not for the animal category, a sex difference that has been demonstrated in cohorts of healthy controls.^27^ We did not identify differences in the rate of decline in neuropsychological performances in the prodromal stage of DLB.

Previous research has established females often have a memory advantage compared to males across the lifespan.^28^, ^29^ Despite females having greater cognitive reserve than males, cognitive decline rates can also be faster for females than males with overt dementia.^28^, ^30^ Thus, while females may perform similarly or better in the prodromal stage of DLB than males, females can experience faster progression of cognitive decline after dementia onset. While non‐amnestic dementia is more likely in DLB than AD, people with DLB can often report memory changes first.^31^, ^32^ Given the heterogeneity of this disorder, detailed and repeat neuropsychological evaluations can help better identify the clinical profile and rate of progression.

Neuropsychiatric symptoms are common throughout the course of DLB. We identified a sex difference for disinhibition 2 years prior to dementia diagnosis, but the clinical relevance is unclear given the small number with this symptom. Disinhibition in DLB has been previously associated with frontal lobe involvement.^33^ Frontal atrophy may be more frequent for males than females with DLB, although this sex difference tends to disappear with age.^34^ The higher frequency of disinhibition for females may be associated with their older age compared to males. In our cohort, we did not identify sex differences for other psychiatric symptoms at first visit with dementia or in the prodromal stage, in contrast to prior research. Depression and auditory hallucinations have also been reported to be more frequent for females than males with DLB at the time of diagnosis.^26^ With advanced dementia, females can experience more severe appetite/eating problems, apathy, and irritability.^35^ Although there is evidence visual hallucinations occurred more frequently and earlier in females than males with DLB in some clinical cohorts, we did not find this in our cohort and this sex difference is not consistently reported in pathology‐confirmed cohorts.^2^, ^36^

We noted a higher frequency of probable RBD for males in the prodromal stage compared to females, consistent with findings in both clinical and pathology‐confirmed DLB cohorts.^2^ Absence of RBD leads to delayed diagnosis of DLB for both females and males, underscoring its importance for the diagnostic approach.^5^ Symptom‐based screening questionnaires for RBD may lead to overdiagnosis in males and underdiagnosis in females.^37^ Video‐polysomnography can help exclude RBD mimics such as non‐REM parasomnias, periodic limb movements during sleep, nocturnal seizures, and obstructive sleep apnea, which is more common in males.^38^

Both clinically defined and pathology‐confirmed cohorts of DLB report higher prevalence of parkinsonism in males than females.^2^ We did not identify sex differences in the percentages of individuals with at least one symptom of parkinsonism. However, specific symptoms of bradykinesia and rigidity were more common in males relative to females in the prodromal phase. Females were more likely to report falls at the first visit with a DLB diagnosis. Notably, we assessed presence of falls rather than frequency or severity, consistent with other prodromal DLB studies.^31^ Females may experience milder parkinsonism than males,^2^ which is not captured in our analysis.[Table alz70275-tbl-0003]

Percentage of females and males experiencing cognitive fluctuations are often reported to be similar in DLB cohorts.^2^ Although we also found similar rates of cognitive fluctuations at the first visit with a DLB diagnosis, we noted a higher percentage of males had cognitive fluctuations than females 2 years prior to diagnosis. This is contradictory to a study in China reporting higher percentages of females with cognitive fluctuations than males in prodromal DLB, without significant sex differences after dementia onset.^39^ In their study, females had lower scores on global cognitive screening tests than males in the prodromal stage, and the authors suggested worse cognitive functioning may contribute to higher rates of cognitive fluctuations.

Dynamic brain network impairment within the ascending arousal system has been suggested to underlie cognitive fluctuations with attention deficits as an early indicator for this symptom.^40^ In our cohort, performances on cognitive screening and attention measures were similar for sexes. For healthy adults, the amount of time at rest spent in attention‐related brain networks and transition patterns from default mode network states into the attention‐related networks differ by sex.^41^ Therefore, it is likely that underlying mechanisms for cognitive fluctuations differ by sex in DLB and need further investigation.

While core features are useful for the clinical diagnosis, cognitive, behavioral, and motor symptoms beyond these core features are also frequent and can differ by sex.^2^, ^42^, ^43^ This may be particularly important for females, as females with DLB have a higher rate of misdiagnosis.^3^, ^6^ Our findings suggest females are less likely than males to experience the core features in the prodromal stage. For instance, severe neuroleptic sensitivity, autonomic symptoms, sleep problems, and visuospatial and executive deficits are more common in DLB in the prodromal stage or after dementia onset compared to AD.^32^, ^42^, ^43^

The current study is one of the first to examine sex differences in prodromal presentations of DLB.^2^, ^26^ The use of the NACC‐UDS is a considerable strength, as this includes one of the largest cohorts of participants with prodromal DLB. The prospectively collected data in NACC‐UDS and the newly added Lewy Body Dementia Module allowed us to investigate numerous clinical symptoms prior to a diagnosis of DLB to better characterize symptoms and inform clinical diagnostic criteria.^20^, ^44^, ^45^ In AD, an interplay of biological (e.g., genetics, sex chromosomes and hormones, immune, inflammatory, cardiovascular pathways) and modifiable risk factors have been suggested to underlie sex differences (e.g., education, physical activity, reproductive health, cardiovascular health, mood disorders).^28^ In DLB, sex‐specific genetic risk factors and reproductive health risk factors have been reported.^46^, ^47^ However, additional research is necessary to provide better insight to the underlying mechanisms for sex differences in DLB development and progression.

There are several limitations in our study. Our analysis included individuals mostly identifying as non‐Hispanic and White with high levels of education, limiting generalizability of the findings. More inclusive cohorts with diverse representation will improve the applicability of future research efforts. Sample size for females was small, limiting the ability to detect true differences in clinical symptoms.

Co‐pathologies influence the clinical phenotype, and AD co‐pathology can occur more frequently for females.^4^, ^6^, ^7^, ^48^ This is an important consideration given the prevalence, severity, and clinical correlations of the underlying AD co‐pathology differs by sex in people with LB pathology.^6^, ^7^, ^8^ Higher levels of AD co‐pathology, more frequent for females, may contribute to cognitive decline, particularly among females.^6^, ^8^ Exclusion of people with clinical or pathological diagnosis of AD likely led to a selection bias in our study considering the sex differences for AD misdiagnosis rates. However, co‐occurring AD and LB pathologies both play a role in the clinical profile,^49^ and we are unable to define which pathology occurred first for more adequate classification. To avoid including people with AD and LB co‐pathology occurring afterward, we defined our study sample based on the clinical diagnostic criteria with high specificity.^50^ In clinical practice, multimodal biomarkers, including fluorodeoxyglucose positron emission tomography, dopamine transfer single‐photo emission computed tomography, and/or cerebrospinal fluid assays may be helpful in identifying co‐pathology within an individual. Disease‐modifying treatments among individuals with biomarker‐confirmed co‐pathology should be investigated to enhance clinical applicability.

The prodromal DLB research criteria highlight three potential phenotypes including (1) cognitive symptom onset, (2) psychiatric onset, and (3) delirium onset.^11^ The cognitive symptom onset phenotype is currently the most widely researched. It is possible there may be differences in rates of delirium or a recent onset of psychiatric symptoms between females and males. Emerging research suggests new‐onset psychosis may be more common among females, but additional studies are needed.^26^ It is also likely some individuals experience symptoms of more than one prodromal phenotype in clinical practice. Unfortunately, it was not possible to examine specific prodromal phenotypes in the current study.

In conclusion, findings from this North American cohort emphasize that sex differences occur in prodromal presentations of DLB. These findings may serve to guide future studies among diverse, global cohorts to improve generalizability. In our sample, males were more likely to demonstrate two or more core clinical features earlier in the prodromal period. Prior to a DLB diagnosis, females were less likely to report symptoms of RBD and cognitive fluctuations. No significant differences in neuropsychiatric symptoms or neuropsychological test performance were identified. This work highlights the need to better define the clinical profile and progression of DLB symptoms for females to improve diagnostic accuracy and personalize treatment plans. Sex‐specific approaches may be helpful to disentangle the clinical heterogeneity in this disorder and identify individuals at risk for DLB.

## CONFLICT OF INTEREST STATEMENT

KW receives research support from the NIH (R21AG074368) and as the local co‐PI of a Lewy Body Dementia Association Research Center of Excellence. TJF receives research support from NIH (U01NS100620, P30AG062677, U19WU220252), and from the Mayo Clinic Dorothy and Harry T. Mangurian Jr. Lewy body dementia program. MJA receives research support from the NIH (R01AG068128, P30AG066506, R01NS121099, R44AG062072), the Florida Department of Health (grants 24A14, 24A15), and as the local PI of a Lewy Body Dementia Association Research Center of Excellence. She serves on the DSMBs for the Alzheimer's Therapeutic Research Institute/Alzheimer's Clinical Trial Consortium and the Alzheimer's Disease Cooperative Study. BP receives research support from the NIH (K23 5K23AG073575). SYC receives research support from the NIH (K23 AG073525‐01A1), and as the local co‐PI of a Lewy Body Dementia Association Research Center of Excellence. EAC has no conflicts to report. MJB receives grant support from the NIH (R21AG077469, R21AG074368) and as the local co‐PI of a Lewy Body Dementia Association Research Center of Excellence EB has no conflicts to report and receives research support from NIH (R00AG073453) and Lewy Body Dementia Association. Author disclosures are available in the supporting information.

## CONSENT STATEMENT

The study was conducted in accordance with the ethical standards as laid down in the 1964 Declaration of Helsinki. Written informed consent was obtained from participants at their respective Alzheimer's Disease Research Center (ADRC) data collection site.

## Supporting information



Supporting Information

Supporting Information
