# Supplementary material for: Sex differences in prodromal dementia with Lewy bodies using the National Alzheimer's Coordinating Center data
Source: Alzheimers Dement. 2025 May 19;21(5):e70275. doi: 10.1002/alz.70275 (PMC12089076; doi:10.1002/alz.70275)
Supplement: Supplementary file 2 — Supporting Information [file ALZ-21-e70275-s002.docx]

**Supplemental Table 1.** Neuropsychiatric Symptom Inventory Questionnaire (NPI-Q) symptoms in males and females

|  | **2 Years Prior** | | | **1 Year Prior** | | | **First Visit with DLB Diagnosis** | | |
| --- | --- | --- | --- | --- | --- | --- | --- | --- | --- |
| Clinical Features | Female (N=14) | Male (N=65) | p-value | Female (N=19) | Male (N=95) | p-value | Female  (N=23) | Male (N=120) | p-value |
| Delusions | 7.1% | 1.5% | .325 | 5.3% | 11.6% | .687 | 26.1% | 21.7% | .597 |
| Hallucinations | 21.4% | 12.3% | .401 | 26.3% | 22.1% | .766 | 34.8% | 35.8% | >.999 |
| Agitation/Aggression | 21.4% | 16.9% | .705 | 21.1% | 21.1% | >.999 | 52.2% | 32.5% | .096 |
| Depression/Dysphoria | 57.1% | 40.0% | .254 | 47.4% | 35.8% | .438 | 47.8% | 44.2% | .821 |
| Anxiety | 35.7% | 35.4% | >.999 | 42.1% | 46.3% | .805 | 34.8% | 51.7% | .174 |
| Elation/Euphoria | 7.1% | 3.1% | .448 | 0.0% | 3.2% | >.999 | 8.7% | 5.0% | .615 |
| Apathy/Indifference | 35.7% | 29.2% | .75 | 42.1% | 49.5% | .621 | 39.1% | 49.2% | .495 |
| Disinhibition | 21.4% | 3.1% | .037* | 15.8% | 12.6% | .714 | 30.4% | 18.3% | .255 |
| Irritability/Lability | 28.6% | 24.6% | .744 | 26.3% | 32.6% | .788 | 39.1% | 40.0% | >.999 |
| Motor Disturbances | 14.3% | 12.3% | >.999 | 21.1% | 8.4% | .113 | 17.4% | 23.3% | .785 |
| Nighttime behaviors | 35.7% | 49.2% | .393 | 47.4% | 53.7% | .626 | 39.1% | 57.5% | .116 |
| Appetite/Eating Problems | 28.6% | 24.6% | .744 | 21.1% | 29.5% | .582 | 21.7% | 30.0% | .615 |

**p*<.05

DLB = Dementia with Lewy bodies
